# Diagnostic and therapeutic challenges of treating opportunistic fungal cellulitis: a case series

**DOI:** 10.1186/s12879-022-07365-8

**Published:** 2022-05-05

**Authors:** Jed Paul, Mary M. Czech, Ramya Balijepally, Janice Wes Brown

**Affiliations:** 1 Department of Medicine, Stanford University School of Medicine, 300 Pasteur Drive, Lane Building, L134 MC:5107, Stanford, CA 94305-5107 USA; 2 Division of Infectious Diseases, Stanford University School of Medicine, Stanford, CA USA; 3 Division of Blood and Marrow Transplantation, Stanford University School of Medicine, Stanford, CA USA

**Keywords:** Fungal cellulitis, *Fusarium solani*, *Purpureocillium lilacinum*, Immunocompromised, Opportunistic infection

## Abstract

**Background:**

Cellulitis is an infection most commonly caused by bacteria and successfully treated with antibiotics. However, certain patient populations, especially the immunocompromised, are at risk for fungal cellulitis, which can be misidentified as bacterial cellulitis and contribute to significant morbidity and mortality.

**Case presentations:**

We describe three cases of opportunistic fungal cellulitis in immunosuppressed patients that were initially mistaken for bacterial infections refractory to antibiotic therapy. However, atypical features of cellulitis ultimately prompted further diagnostics to identify fungal cellulitis and allow initiation of appropriate antifungals. We discuss: (1) a 52-year-old male immunosuppressed hematopoietic cell transplant recipient with *Fusarium solani* cellulitis on his right lower extremity that was treated with amphotericin B and voriconazole with full resolution of the cellulitis; (2) a 70-year-old male lung transplant recipient with *Fusarium solani* cellulitis on his left lower extremity that ultimately progressed despite antifungals; and (3) a 68-year-old male with a history of kidney transplantation with suspected *Purpureocillium lilacinum* cellulitis on his left lower extremity ultimately treated with posaconazole with resolution of the skin lesions.

**Conclusions:**

*Fusarium solani* and *Purpureocillium lilacinum* are important pathogens causing opportunistic fungal cellulitis. These cases remind providers to be vigilant for fungal cellulitis when skin and soft tissue infection does not adequately respond to antibiotics and atypical features of cellulitis are present.

## Background

Cellulitis is a common infection, with incidence estimated at around 200 cases per 100,000 person-years in the USA [1]. The vast majority of cases are caused by bacterial etiologies such as *Streptococcus* and *Staphylococcus*, and less commonly gram-negative bacteria, with over 95% of cases responding to antimicrobial monotherapy. However, in immunocompromised patients, and rarely in immunocompetent hosts, cellulitis may be due to fungi. Because of the relative rarity of fungal skin and soft tissue infection, misidentification of these cases often leads to inappropriate treatment and significant morbidity or mortality [1, 2]. In this paper, we highlight three cases of fungal cellulitis that were initially mistaken for bacterial cellulitis in part due to modest response to antibacterial therapy, and discuss the importance of fungal consideration when atypical features of cellulitis are identified.

## Case presentation

### Case 1

**Fig. 1:**
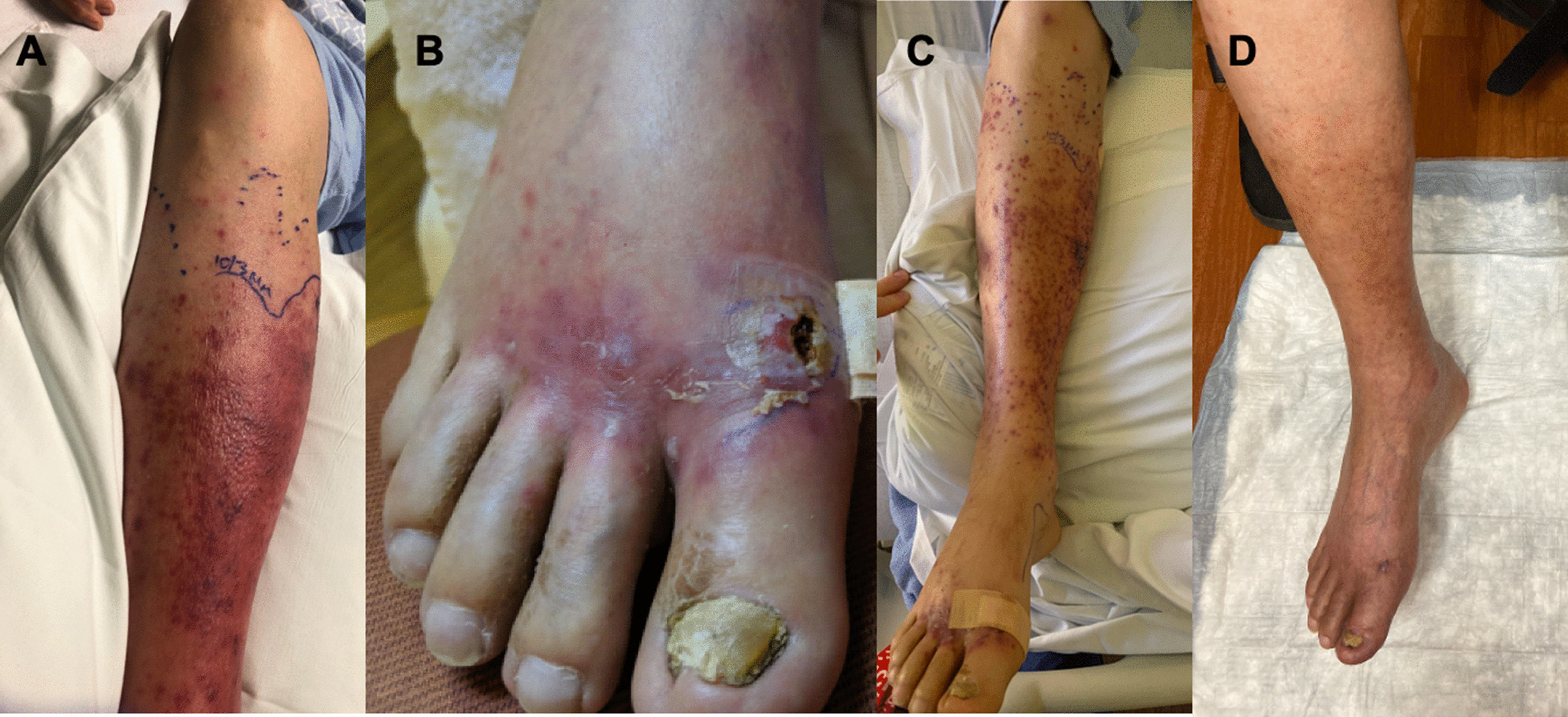
Patient 1’s leg and foot on presentation (**A**, **B**), partial response after antibiotic therapy (**C**), and three months post-discharge after antifungal therapy (**D**)

A 52-year-old male with insulin-dependent type 2 diabetes (hemoglobin A1C 9.2%) and a history of B cell acute lymphoblastic leukemia, who received an allogeneic hematopoietic cell transplant (HCT) four years prior to presentation, presented with right lower extremity pain and erythema. HCT was complicated by chronic graft-versus-host disease of the skin and oral mucosa for which the patient was maintained on prednisone 10 mg daily and ruxolitinib 10 mg twice daily. Five days prior to presentation, he noticed a small, painful papule on the dorsal hallux of his right foot which became ulcerative with painful erythema that spread circumferentially over his calf. He was prescribed a course of clindamycin with transient improvement. On admission, in addition to the erythema and right hallux ulcer, he was noted to have an onychomycotic right first toe (Fig. 1). He was started on broad spectrum antimicrobial coverage with intravenous (IV) vancomycin 1.25 g twice daily, meropenem 1 g three times daily, and isavuconazonium 4 mg/kg daily with improvement in his dermatitis over the next several days. A wound culture from the ulcer grew *Fusarium solani*, with pathology showing dermal edema with interstitial neutrophilic and lymphohistiocytic infiltrates. Polymerase chain reaction testing and fungal culture of the onychomycotic toenail additionally showed *Fusarium solani*, suggesting that cellulitis resulted from extension of the infected toenail. For *Fusarium solani* cellulitis, isavuconazonium was discontinued and both liposomal amphotericin B 5 mg/kg daily and intravenous voriconazole 4 mg/kg twice daily after a 6 mg/kg loading dose were started on hospital day 8. He was discharged home on hospital day 16 with residual but improving erythema of the leg on an additional four weeks of liposomal amphotericin B and voriconazole plus topical efinaconazole for his affected nail. He continued to improve over the ensuing months. Ultimately, he had only minimal scarring from his ulcer ten months after discharge alongside a persistently onychomycotic but improved toenail.

### Case 2

**Fig. 2:**
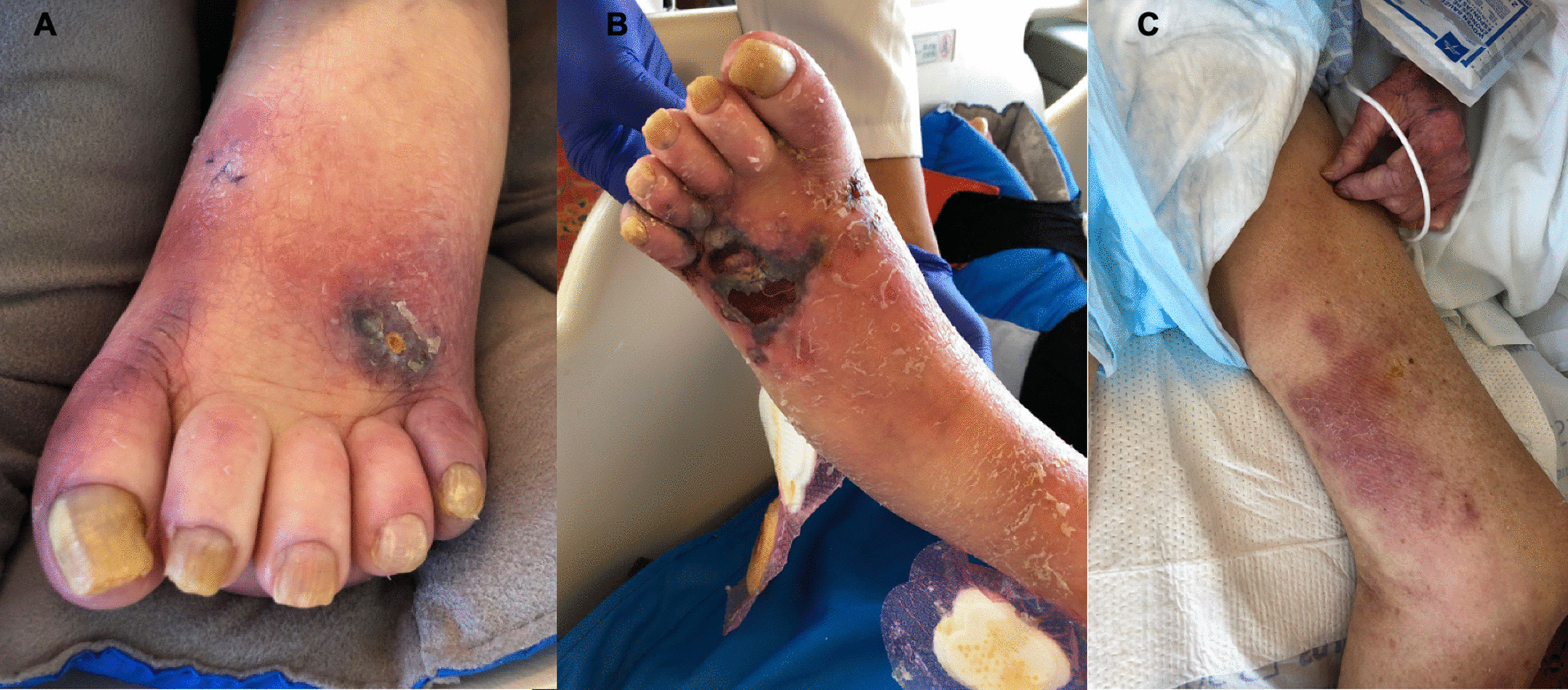
Patient 2’s left foot when lesion was first noticed on post-transplant day 86 (**A**) and progression despite antibiotic and antifungal therapy on post-transplant day 103 (**B**) with spread to thigh (**C**)

A 70-year-old male with a history of scleroderma-induced interstitial pneumonia, who underwent bilateral lung transplantation, developed left lower extremity cellulitis two months following his transplant. His post-transplant course was complicated by prolonged hospitalization for chronic mechanical respiratory failure unrelated to allograft function and multiple cardiac arrests. He received induction immunosuppression with basiliximab, and at the time of cellulitis he was maintained on azathioprine 50 mg daily and prednisone 5 mg twice daily. He received 5 days of IV cefazolin with improvement, but the erythema returned after antibiotics were stopped. Four days later, he was started on IV vancomycin 750 mg daily and cefepime 1 g daily (renal dosing). However, his erythema persisted, and he developed a black, ulcerative lesion on the dorsum of his left foot, in the setting of multiple onychomycotic toenails (Fig. 2). After nine days on vancomycin and cefepime, skin biopsy cultures grew *Fusarium solani*. Corresponding pathology of the biopsy specimen showed deep dermal fungal elements. He was started on IV amphotericin B 5 mg/kg daily and IV voriconazole 4 mg/kg twice daily after two loading doses. Blood cultures, respiratory cultures, chest computed tomography (CT) scan, and ophthalmologic exam were unrevealing for disseminated fungal infection. Although samples from the onychomycotic toenails were not evaluated, it is suspected that onychomycosis was due to *Fusarium*, and skin breakdown surrounding the nail allowed for fungal inoculation. Despite antifungal therapy, the patient developed progressive erythematous nodules extending proximally to his upper thigh, consistent with vascular spread of infection. He subsequently developed septic shock refractory to broad spectrum antibiotics and ongoing antifungals. He was transitioned to comfort care and expired on post-transplant day 113.

### Case 3

**Fig. 3:**
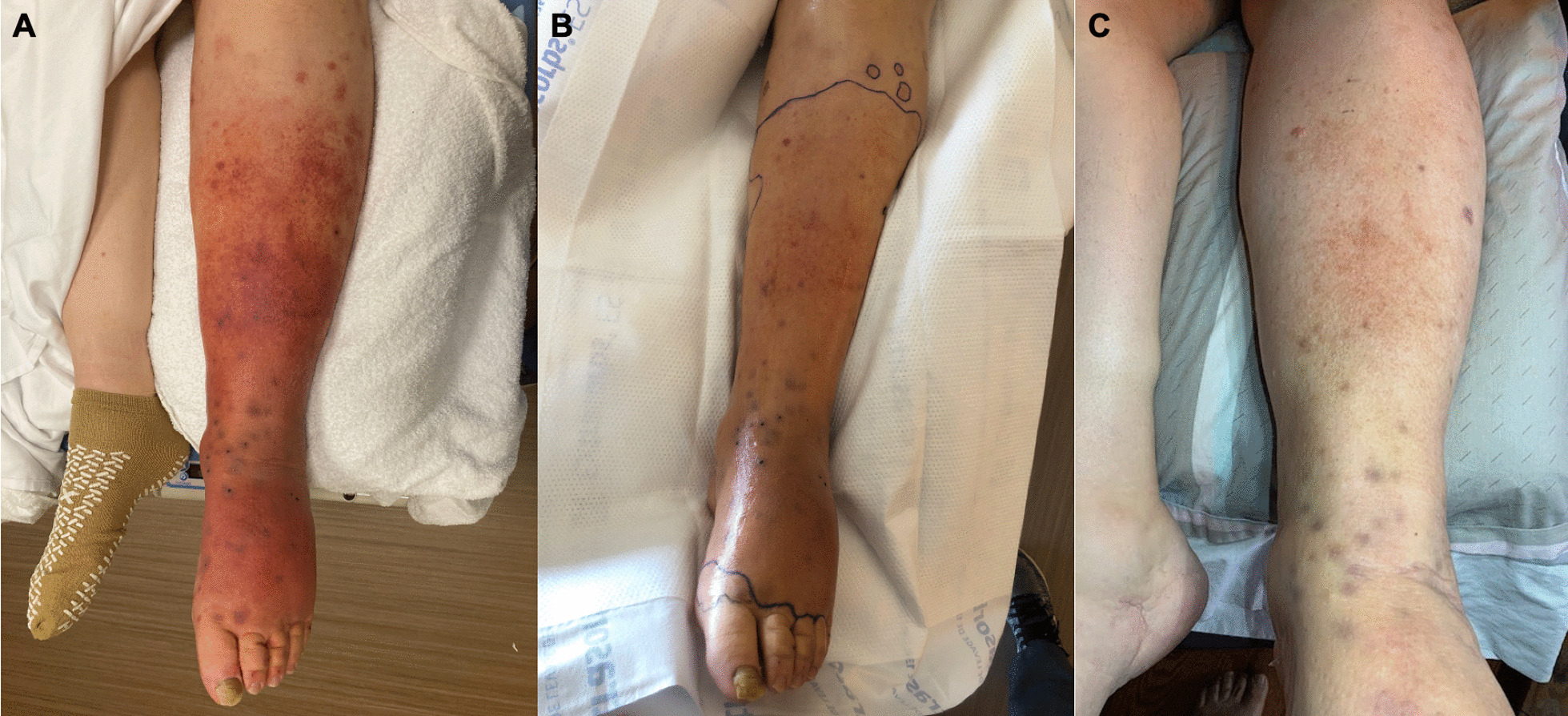
Patient 3’s leg on presentation (**A**), after partial response to IV antibiotics (**B**), and one month after discharge on systemic antifungal therapy (**C**)

A 68-year-old male with a history of Alport Syndrome, who received a living related donor kidney transplant 30 years prior to presentation, presented with a 9-day history of global left lower extremity swelling with distal erythema and pain. His immunosuppressive regimen was azathioprine 100 mg daily and tacrolimus 2 mg daily. He had a recent folliculitis in his left groin 3 weeks prior to presentation, treated with ten days of oral doxycycline and fourteen days of oral azithromycin. Two weeks following the folliculitis, he was admitted at an outside hospital with new fever, progressive left lower extremity erythema, swelling and pain consistent with cellulitis. Within 48 h of starting IV vancomycin and clindamycin for presumed Methicillin-resistant *Staphylococcus aureus* (MRSA) cellulitis, fevers resolved and erythema and swelling improved. However, after discharge, his cellulitis again worsened, and he developed exquisite leg tenderness with the development of multiple 2–4 mm tender purplish-black papules, some with punctate central eschar. On presentation to our hospital, these skin changes were noted to occur in the setting of longstanding first toe onychomycosis (Fig. 3). He was started on IV cefepime 2 g twice daily (renal dosing) and daptomycin 800 mg daily with marked improvement in both his erythema and pain over the next few days. However, the violaceous papules showed no change, prompting a punch biopsy of one papule. Gram stain of the biopsy specimen was unrevealing for significant polymorphonuclear cells (PMNs) or organisms. Tissue culture was negative for both bacterial and fungal growth. However, given concern for biopsy sampling error, he was started on empiric oral itraconazole 200 mg twice daily for presumed fungal cellulitis. Subsequently, fungal polymerase chain reaction of the onychomycotic toenail identified *Purpureocillium lilacinum*, which was also believed to be the causative pathogen of the purplish-black cutaneous papules. There was no evidence of deep tissue infection on magnetic resonance imaging (MRI) of his leg, and the CT scan of his chest did not suggest evidence of disseminated fungal infection. His pain and swelling improved by hospital day four, and he was discharged home on hospital day seven with fourteen additional days of doxycycline 100 mg twice daily and levofloxacin 750 mg every other day (renal dosing), as well as a prolonged course of itraconazole 200 mg twice daily. On follow-up 15 days after discharge, his erythema, swelling, and pain had resolved. Following 12 weeks of itraconazole, the black papules had flattened and faded, at which time antifungal therapy was stopped. However, one month after discontinuation of itraconazole the papular lesions reappeared with new lesions in the same distribution of his left leg. He was retreated with posaconazole, and three months into therapy, the black papules resolved.

## Discussion and conclusions

These cases demonstrate episodes of fungal cellulitis initially mistaken for pure bacterial cellulitis in immunosuppressed patients. In all cases, summarized in Table 1, patients were initially treated with antibacterial agents with at least partial response to therapy. However, when the episodes of cellulitis seemed refractory to appropriate antibiotics, and displayed atypical features of cellulitis, opportunistic infections were appropriately considered. Diagnosis ultimately required clinical suspicion with tissue biopsy, cultures, and pathology to diagnose fungal cellulitis. With an accurate diagnosis, patients were able to be started on targeted long term antifungal therapy.

**Table 1:** Summary of case reports

	Underlying condition	Immuno- suppression	Atypical features of cellulitis	Fungal Pathogen	Antifungal regimen	Outcome	Relevant citations for other related case reports
Patient 1	B cell ALL s/p allo-HSCT c/b cGVHD	Prednisone 10 mg daily, ruxolitinib 10 mg twice daily	Ulcerative lesion on dorsum of foot unresponsive to antibiotics	*Fusarium* solani	5 weeks of IV amphotericin B 5 mg/kg daily and voriconazole 4 mg/kg twice daily	Near baseline (residual scarring)	[4, 5, 18, 19, 22]
Patient 2	Systemic scleroderma c/b interstitial pneumonia s/p bilateral lung transplant	Azathioprine 50 mg daily, prednisone 5 mg twice daily	Ulcerative lesion on dorsum of foot unresponsive to antibiotics	*Fusarium* solani	Amphotericin B 5 mg/kg daily and voriconazole 4 mg/kg twice daily	Deceased (refractory septic shock)	[4, 5, 18, 19, 23]
Patient 3	Alport Syndrome c/b ESRD s/p kidney transplant	Azathioprine 100 mg daily, tacrolimus 2 mg daily	Numerous violaceous papules on calf and dorsum of foot unresponsive to antibiotics	*Purpureocillium* lilacinum	8 weeks of itraconazole 200 mg twice daily	Recurrence after discontinuation of itraconazole, responded to posaconazole	[3, 6, 9, 12, 13, 18, 19]

*ALL* acute lymphoblastic leukemia, *s/p* status post, *allo-HCT* allogeneic hematopoietic cell transplantation, *c/b* complicated by, *cGVHD* chronic graft versus host disease, *ESRD* end-stage renal disease

Fungal cellulitis has been increasingly described in immunocompromised individuals [3–6]. Impaired functioning of cell-mediated immunity pathways, particularly those involving T cells, predispose immunocompromised patients to infection with opportunistic and environmental fungi [7, 8]. As described in these cases, *Fusarium solani* and *Paecilomyces lilacinus*, renamed to *Purpureocillium lilacinum*, are ubiquitous fungi found in soil and decaying vegetation [9, 10]. As with most fungi, infection commonly occurs via inhalation with subsequent dissemination to skin and other sites, or as direct inoculation, often at a site of skin or mucosal barrier breakdown [5, 11]. Cutaneous infection with these fungi manifests in many forms, including painless papules, tender nodules, and necrotic ulcers, occasionally requiring surgical debridement in addition to systemic antifungal therapies [3, 5, 12, 13].

Diagnosis of fungal cellulitis poses a significant challenge to clinicians for multiple reasons. As seen in our cases, fungal infections may mimic or possibly co-occur with bacterial infections, and a partial response to antibiotic therapy can give a false impression of adequate antimicrobial coverage. This prolongs the treatment course and puts severely immunocompromised patients at risk for both more severe local disease as well as disseminated fungal infection. Furthermore, the broad spectrum of manifestations of skin and soft tissue infections, especially among the immunocompromised, leads to misidentification of fungal lesions. Finally, even if a biopsy is obtained, advanced testing modalities may be required, and results must be interpreted with caution and consideration for the clinical context. For example, patient 3’s skin biopsy was largely unrevealing. However, the absence of PMNs in gram stain was highly suggestive of sampling error given clear evidence of inflammation on exam and the absence of neutropenia in the patient’s peripheral blood [14]. Therefore, clinicians were prompted to evaluate the patient’s onychomycotic toenail, which was positive for *P. lilacinum*. Though it is possible different organisms caused the skin and toenail infections, we suspect the scattered necrotic papules resulted from *P. lilacinum* cutaneous infection tracking from the toenail.

To aid in diagnosis, differences in the presentation of bacterial and fungal cellulitis may help clinicians to more readily identify atypical and fungal cellulitis. Typical bacterial cellulitis commonly presents with erythema, swelling, and pain, which rapidly improves with antimicrobial therapy. For typical bacterial cellulitis, open wounds often serve as entry portals for bacteria to cause infection, but less commonly do open wounds develop as a result of infection (some exceptions being MRSA, *Streptococcus pyogenes*, and polymicrobial necrotizing fasciitis). For patients 1 and 2, they developed open wounds on the dorsum of the foot. As discussed, this is unusual for typical bacterial cellulitis, and furthermore an unusual location for a traumatic wound. This unusual feature prompted biopsy consistent with fungal infection, which commonly causes ulceration due to invasion of the vascular supply. Additionally, for patient 3, scattered black papules are an unusual feature of bacterial cellulitis, which appropriately prompted further investigation.

Ultimately, tissue is often required for correct identification of fungal pathogens by either standard microbiological or molecular techniques. Identification of fungal pathogens to the species level is essential for selection of appropriate therapy. Although there are no established breakpoints for antifungal susceptibility testing against filamentous fungi, there is often a body of literature to help guide the choice of therapeutics. For example, *F. solani* infections typically respond to combination therapy with amphotericin B and voriconazole, though successful therapy with posaconazole or isavuconazole have also been described [5]. *P. lilacinum* can have some degree of intrinsic *in vitro* resistance to several commonly used mold active agents, such as amphotericin products, itraconazole, and echinocandins, but favorable minimum inhibitory concentrations for voriconazole and posaconazole [15–17]. Given that clinical outcomes have not been well defined for minimal inhibitory or fungicidal concentrations to establish breakpoints, clinicians should closely monitor patients being treated for fungal cellulitis to ensure appropriate clinical response.

Onychomycosis is a common feature in bacterial and fungal cellulitis and has widely been documented as a risk factor for development of cellulitis [18–20]. The exact mechanism behind this association is unclear. However, other reports describe onychomycosis with either paronychia or interdigital intertrigo serving as a suspected cutaneous portal of entry for invasive fungal infection [21]. Given the proximity of all our patients’ cellulitis to preexisting onychomycotic infections, we similarly suspect onychomycosis with surrounding skin breakdown led to extension of infection resulting in cellulitis.

These cases highlight the importance of consideration for fungal pathogens when cellulitis displays atypical features in immunocompromised hosts. Prolonged treatment targeted at only bacterial pathogens can potentially lead to significant morbidity or mortality when fungal cellulitis is present. Clinicians should be mindful of these atypical features of cellulitis and investigate possible fungal etiologies in warranted cases.

## Data Availability

All relevant data is included in the manuscript. A separate data repository is not applicable.

## Abbreviations

HCT: Allogeneic hematopoietic cell transplant
IV: Intravenous
CT: Computed tomography
MRSA: Methicillin-resistant *Staphylococcus aureus*
ESR: Erythrocyte sedimentation rate
CRP: C-reactive protein
PMNs: Polymorphonuclear cells
MRI: Magnetic resonance imaging
